# Supplementary material for: Multi‐dataset identification of innovative feature genes and molecular mechanisms in keratoconus
Source: J Cell Mol Med. 2024 Sep 19;28(18):e70079. doi: 10.1111/jcmm.70079 (PMC11412914; doi:10.1111/jcmm.70079)
Supplement: Supplementary file 2 — Material S2. [file JCMM-28-e70079-s002.zip › 7.GSEA analysis/3.DBNDD1/KEGG_LYSINE_DEGRADATION.html]

Details for gene set KEGG\_LYSINE\_DEGRADATION[GSEA]

|  || Dataset | DBNDD1.group.cls#h\_versus\_l |
| Phenotype | group.cls#h\_versus\_l |
| Upregulated in class | l |
| GeneSet | KEGG\_LYSINE\_DEGRADATION |
| Enrichment Score (ES) | -0.36986023 |
| Normalized Enrichment Score (NES) | -1.1883727 |
| Nominal p-value | 0.3108911 |
| FDR q-value | 0.7354524 |
| FWER p-Value | 0.99 |
Table: GSEA Results Summary

  

Fig 1: Enrichment plot: KEGG\_LYSINE\_DEGRADATION      
 Profile of the Running ES Score & Positions of GeneSet Members on the Rank Ordered List

  

| SYMBOL | TITLE | RANK IN GENE LIST | RANK METRIC SCORE | RUNNING ES | CORE ENRICHMENT || 1 | OGDHL | na | 6051 | 0.149 | -0.1272 | No |
| 2 | TMLHE | na | 7309 | 0.118 | -0.1262 | No |
| 3 | NSD1 | na | 8639 | 0.092 | -0.1354 | No |
| 4 | BBOX1 | na | 9554 | 0.077 | -0.1373 | No |
| 5 | ASH1L | na | 10289 | 0.067 | -0.1371 | No |
| 6 | AASDH | na | 10637 | 0.063 | -0.1273 | No |
| 7 | DLST | na | 12361 | 0.044 | -0.1631 | No |
| 8 | AASDHPPT | na | 12757 | 0.040 | -0.1617 | No |
| 9 | ALDH3A2 | na | 12835 | 0.040 | -0.1515 | No |
| 10 | ECHS1 | na | 14374 | 0.027 | -0.1871 | No |
| 11 | ACAT1 | na | 14381 | 0.027 | -0.1787 | No |
| 12 | ACAT2 | na | 15183 | 0.021 | -0.1950 | No |
| 13 | SETMAR | na | 16878 | 0.010 | -0.2406 | No |
| 14 | HADHA | na | 17010 | 0.009 | -0.2414 | No |
| 15 | EHMT1 | na | 17366 | 0.007 | -0.2494 | No |
| 16 | HADH | na | 17746 | 0.005 | -0.2588 | No |
| 17 | SETDB2 | na | 18227 | 0.001 | -0.2723 | No |
| 18 | ALDH7A1 | na | 18637 | -0.002 | -0.2835 | No |
| 19 | SUV39H1 | na | 19725 | -0.011 | -0.3115 | No |
| 20 | GCDH | na | 20439 | -0.017 | -0.3267 | No |
| 21 | SETD7 | na | 20868 | -0.021 | -0.3325 | No |
| 22 | SETD1A | na | 21001 | -0.022 | -0.3293 | No |
| 23 | ALDH1B1 | na | 22410 | -0.036 | -0.3584 | Yes |
| 24 | ALDH9A1 | na | 22786 | -0.041 | -0.3563 | Yes |
| 25 | DOT1L | na | 22993 | -0.043 | -0.3487 | Yes |
| 26 | SUV39H2 | na | 23008 | -0.043 | -0.3355 | Yes |
| 27 | SETD1B | na | 23043 | -0.044 | -0.3227 | Yes |
| 28 | EHMT2 | na | 24057 | -0.057 | -0.3338 | Yes |
| 29 | PIPOX | na | 24187 | -0.059 | -0.3190 | Yes |
| 30 | SETDB1 | na | 25624 | -0.081 | -0.3348 | Yes |
| 31 | SETD2 | na | 25760 | -0.083 | -0.3124 | Yes |
| 32 | OGDH | na | 26258 | -0.093 | -0.2974 | Yes |
| 33 | PLOD3 | na | 27796 | -0.126 | -0.3017 | Yes |
| 34 | PLOD2 | na | 28562 | -0.145 | -0.2779 | Yes |
| 35 | EHHADH | na | 30010 | -0.189 | -0.2598 | Yes |
| 36 | AADAT | na | 30840 | -0.215 | -0.2155 | Yes |
| 37 | PLOD1 | na | 31475 | -0.239 | -0.1581 | Yes |
| 38 | ALDH2 | na | 33235 | -0.347 | -0.0989 | Yes |
| 39 | AASS | na | 34134 | -0.448 | 0.0171 | Yes |
Table: GSEA details [plain text format]

  

Fig 2: KEGG\_LYSINE\_DEGRADATION      
 Blue-Pink O' Gram in the Space of the Analyzed GeneSet

  

Fig 3: KEGG\_LYSINE\_DEGRADATION: Random ES distribution      
 Gene set null distribution of ES for **KEGG\_LYSINE\_DEGRADATION**
